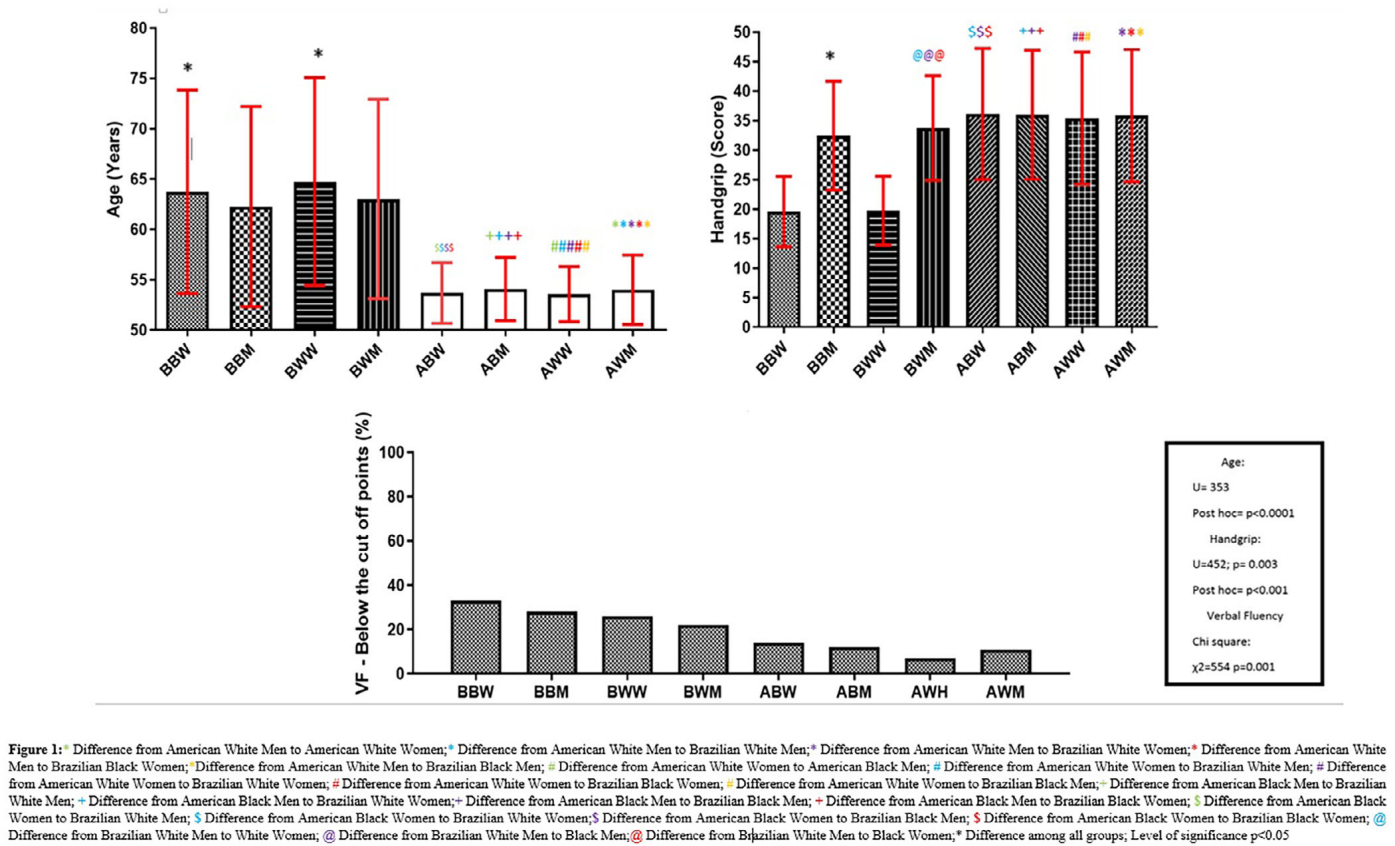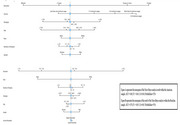# Association between strength levels and cognitive status in Brazilian and Americans older adults: the role of race inequalities

**DOI:** 10.1002/alz70860_103186

**Published:** 2025-12-23

**Authors:** Jessica dos Santos Sacramento Plácido

**Affiliations:** ^1^ Federal University of Rio de Janeiro, Rio de Janeiro, Rio de Janeiro, Brazil

## Abstract

**Background:**

Both Brazil and the United States have histories shaped by slavery and racial segregation, resulting in disparities in healthcare access, particularly for older adults. Black older adults in both countries are at higher risk of cognitive impairment and dementia and face higher rates of underdiagnosis compared to Whites. Key initiatives such as the WHO's Decade of Healthy Aging (2021‐2030) and the UN's Sustainable Development Agenda (2030) call to reduce inequalities and promote healthy aging. However, studies on the impact of racism on health, particularly those considering intersectionality and focusing on older adults, remain limited. This study investigates how the association between strength levels and cognitive impairment in older adults is influenced by racial, gender, and income inequalities in societies shaped by racial segregation.

**Method:**

This cross‐sectional study used baseline data from the Brazilian Longitudinal Study of Aging (ELSI‐Brazil) and the Health and Retirement Study (USA). Strength levels were assessed using handgrip strength, and cognitive status was evaluated with the Verbal Fluency test (VF). Race/color was self‐reported. Data from the 2015 and 2016 cohorts were analyzed using comparative tests, logistic regression models, and machine learning techniques to explore how race, income, gender, and health status mediate the relationship between strength and cognitive performance in both countries.

**Result:**

Older Black adults in both countries showed worse VF scores compared to Whites (Figure 1). Post‐hoc analysis revealed no differences in dynamometry values between White and Black older adults in the USA, but differences were observed in the Brazilian cohort. Brazilian Black adults had almost three times the risk of cognitive impairment on the VF test compared to White Americans (Table 1). Lower handgrip values and older age significantly increased the risk of cognitive impairment. No increased risks were found in the American cohort based on race or gender. Naïve Bayes analysis indicated that being a Black woman with low education, income, strength, and more than two chronic diseases is associated with over 52% probability of cognitive impairment (Figure 2)

**Conclusion:**

Handgrip strength is more strongly associated with cognitive levels in Brazilian older adults. Brazilian Black women have a higher risk of cognitive impairment on the VF test compared to their American counterparts.